# Effect of Hydrothermal Aging and Beverages on Color Stability of Lithium Disilicate and Zirconia Based Ceramics

**DOI:** 10.3390/medicina55110749

**Published:** 2019-11-19

**Authors:** Satheesh B. Haralur, Noura Raqe S. Alqahtani, Fatimah Alhassan Mujayri

**Affiliations:** Department of Prosthodontics, College of Dentistry, King Khalid University, Abha 62529, Saudi Arabia; norah14141@outlook.sa (N.R.S.A.); Dr.fatimh@hotmail.com (F.A.M.)

**Keywords:** lithium disilicate, monolithic zirconia, colour stability, discolouring agents, all ceramic prosthesis

## Abstract

*Background and Objectives*: All-ceramic prosthesis is widely used in modern dental practice because of its improved physico-mechanical and optical properties. These restorations are exposed to coloring agents from various nutrition and beverages in the oral cavity. Long-term color stability is critical for the success of these restorative materials. The purpose of this in vitro study was to assess the effect of common beverages and mouthwash on the color stability of lithium disilicate (LD), monolithic zirconia (MZ) and bilayer zirconia (BZ) surfaces. *Materials and Method*: Thirty disc-shaped specimens from each material were fabricated; each group was subdivided (*n* = 10) according to coffee, green tea and chlorhexidine immersion solutions. The baseline color of ceramic discs was recorded according to the CIE L*a*b* system with a portable spectrophotometer. The second measurement was recorded after 3000 thermocycling and immersion in coloring agents for 7 days. The mean color difference was calculated and data were compared with Kruskal-Wallis and Mann-Whitney post hoc tests (0.05). *Results*: ΔE values for LD with the immersion of coffee, tea, and Chlorhexidine gluconate (CHG) were 1.78, 2.241 and 1.58, respectively. Corresponding ΔE values for MZ were 5.60, 5.19, and 4.86; marginally higher than the clinically acceptable level of 3.5. Meanwhile, BZ showed better color stability compared to MZ with ΔE values of 4.22, 2.11 and 1.43. *Conclusions*: Among the ceramics evaluated, LD ceramic was found to be more color stable, while MZ ceramics displayed a higher susceptibility to discoloration. MZ and BZ ceramic colors were significantly altered with coffee immersion, while LD ceramics were more affected by green tea.

## 1. Introduction

The demand for aesthetic dental services is on a constant upsurge due to an aesthetic conscious society [1]. Aesthetic dental practice has brought numerous innovative clinical procedures and a revolution in dental materials. Indirect restoration constructed from various all-ceramic materials is widely practiced in contemporary dental practice. Lithium disilicate (LD) is the commonly used glass-ceramic because of its exceptional optical properties, superior strength and ease of fabrication [2,3]. Additional advantages of lithium disilicate include better marginal integrity, less porosity and net-shaped forming by pressing [4,5]. When LD prosthesis is fabricated in full contour, it prevents the challenge of physical-mechanical compatibility between two dissimilar materials. Hence it is less likely to crack or for the veneer to crack, when compared to bilayer ceramic restoration. Though LD is one of the versatile indirect restorative materials, care should be exercised in restoring bruxism patients with high occlusal stress and non-vital teeth due to its fracture toughness of 2.8–3.5 MPa [6]. Clinical indications include the tooth-implant supported single crowns, anterior fixed prosthesis, anterior veneers and posterior inlay/onlay [7,8].The search for a material that combines both mechanical properties, such as the resistance offered by metal restoration, and the typical optical properties of glass-ceramic, has led to the introduction of Yttrium stabilized trigonal zirconia polycrystalline (Y-TZP) ceramics. The main disadvantage is its brittle veneering ceramics for debonding, fractures and chipping. These clinical complications led to the rapid evolution of modified microstructure and translucency.

Prosthesis must not only have the dimensions, texture and contours of the teeth to be replaced but should also have similar light behavior. Furthermore, the color stability of the restoration is critical for the long-term success of the aesthetic restorations. Although the physicomechanical properties of ceramics have been vastly improved, they are susceptible to discoloration [9,10]. Extrinsic factors like beverages, mouthwashes, acid solutions, tooth brushing and higher temperatures are reported to induce the surface degradation of ceramics [11,12,13]. The extrinsic pigment absorption or adsorption from the oral cavity is affected by the composition and surface morphology of ceramic materials [14,15]. Various mouthwash agents like chlorhexidine gluconate and benzydamine have antimicrobial properties that are advocated to supplement mechanical oral hygiene methods. The prolonged use of chlorhexidine is known to cause brown staining on teeth, various restorative materials and the dorsum of the tongue. The non-enzymatic browning and formation of pigmented metal sulfide are attributed to the discoloration of teeth and restorations [16]. In the protracted use of chlorhexidine, an increase in the formation of supragingival calculi is also observed [17]. Few researchers have reported that the discoloration is due to the precipitation of the dietary chromogens together with the locally adsorbed chlorhexidine. Previous research reveals that improper color match and color instabilities constitute the main reasons for the clinical replacement of anterior restorations [18]. Studies assessing the discoloration different all-ceramic restoration materials due to the combined effect of hydrothermal aging and commonly consumed beverages and mouthwash are sparse. Therefore, the present study was performed to evaluate the discoloration of Lithium Disilicate, Monolithic and Bilayer Zirconia after in vitro accelerated aging in coffee, green tea and chlorhexidine. The null hypothesis was that the hydrodynamic aging, along with immersion in coffee, tea and chlorhexidine, would have no effect on the color stability of the ceramics.

## 2. Materials and Methods

### 2.1. Specimens’ Preparation

Lithium disilicate specimens were fabricated by the burning out of a 2 mm thickness, 10 mm diameter pattern inlay in wax. These wax patterns were heat pressed with an IPS Empress, Programat, EP 5000 furnace (Ivoclar Vivadent AG Schaan, Liechtenstein) at 920 °C. To finish and flatten the specimen surface, 400–1200 grit silicone carbide papers were used. The specimens were cleaned in an ultrasonic cleaner with distilled water for 10 minutes and were coated with a single layer of the neutral shade glaze by firing at 765 °C.

Monolithic zirconia discs measuring 10 mm in diameter and 2 mm in thickness were fabricated by scanning (D 900, 3Shape North America. New Jersey, USA) the identical size resin pattern. The pre-sintered zirconia block (Dental Direkt GmbH, Spenge, Germany) was copy milled with computer-aided design and a manufacturing system (Cyanoprod, Canada). Milled discs were polished with sequential use of 400–1200 grit silicon carbide papers under a water jet. The milled discs were sintered by further firing at 1450 °C (Nabertherm GmbH, Lilienthal, Germany). The Core specimens for bilayer zirconia discs (Zir prime, Kurary Noritake Dental Inc, Miyoshi Aichi, Japan) were fabricated with yttria-stabilized tetragonal zirconia polycrystal (Y-TZP) by the copy milling of resin pattern following a similar process to that of monolithic zirconia (MZ). One side of the zirconia core was covered with the feldspathic porcelain veneer (Vita VM9, Zahnfabrik H. Rauter GmbH, Bad Sackingen, Germany) with a layering technique following the manufacturer’s instructions. The thickness of the veneering porcelain was kept at 1mm for all the samples and it was confirmed with a digital caliper (FINO GmbH, Mangelsfeld 18, Germany).

### 2.2. Staining Process

The samples from each ceramic material were randomly divided into three subgroup groups of 10. The sample size for each subgroup (*n* = 10) was calculated from the ΔE values of previous studies [9,19], α = 0.05 and power (1-β) = 80%. Each group was divided into 3 subgroups depending on the staining solutions (Chlorhexidine gluconate (CHG), Coffee, and Green tea). The ceramic samples were subjected to thermocycling for 3000 cycles (1100; SD Mechatronik) in distilled water at an alternating temperature of between 5 °C and 55 °C. The coffee (Nescafe Classic, Nestle Middle East manufacturing LLC, Dubai) and tea (Lipton tea; Unilever Gulf FZE, Dubai) staining solutions were prepared by adding 15 g to 250 ml of boiled distilled water. For the purpose of immersion, 0.2% chlorhexidine digluconate oral mouthwash (Corsodyl, GSK, Brentford, Middlesex, UK) was used. Each ceramic disc was immersed within 15 ml of test solution for 7 days at a controlled temperature of 37 ± 10 °C in a dark environment. The test solutions were changed daily and were stirred once every 12 hours to maintain the homogeneity of the solution. After the immersion period of 7 days, the specimens were removed from the testing solution. They were rinsed with distilled water and blot dried with tissue papers. 

### 2.3. Color Measurements

The color of each specimen was recorded before and after thermocycling and immersion within the testing solutions. The color of each sample at both intervals was measured at the identical position (disc center) with a portable spectrophotometer (Vita Easy shade, Vita Zahnfabrik H. Rauter GmbH, Bad Sackingen, Germany). The putty index was made over the disc with a window of 3 mm diameter in the center to standardize the area color measurement. The borders of the window were well-formed with sharp edges. On each occasion, the disc color was measured three times. The average value from 3 repeated color measurements was considered the color of the disc. Three color parameters—‘ L’, ‘a’, ‘b’—were recorded for each ceramic veneer following the CieLab color system. The mean color difference because of immersion of the sample was determined by adopting the formula [20]: ΔE* = ((ΔL*)2 + (Δa*)2 + (Δb*)2) × 1/2, where ΔL* is the variation of L*, Δa* is the variation of a*, and Δb* is the variation of b*. A low ΔE* was considered better shade matching; a score of ≥3.5 was considered acceptable [21].

### 2.4. Statistical Analysis

The statistical analysis was performed using SPSS 19 (IBM Corp, Armonk, NY, USA). Data were analyzed by Kruskal-Wallis and Mann-Whitney post-hoc comparison at the significance level *p* = 0.05.

## 3. Results

The mean L, a, and b values along with the mean color change (ΔE) for all the tested material after immersion in the discoloring solutions are summarized in Table 1. After immersing the ceramic samples in a coffee solution, we observed the highest change in mean color in MZ (5.602), followed by bilayer zirconia (BZ) (4.229) and the LD group (1.788). Green tea immersion also resulted in maximum discoloration of MZ (5.192), the least ΔE value of 2.11 was recorded by BZ. The chlorhexidine led to the least discoloration of all tested groups, ΔE values of 1.588, 4.866 and 1.438 were observed in LD, MZ and BZ groups, respectively. Table 2 demonstrates the results of the Kruskal-Wallis analysis of mean color change between the tested all-ceramic restorative materials. There was a significant difference in the mean ΔE between the groups with coffee immersion; the recorded H value was 19.886 with a *p* value of 0.000. The lower mean rank of 6.10 was recorded by LD and a higher mean rank by MZ (23.40). Immersion in green tea also demonstrated a significant difference between the groups with P-0.007 and H value 10.025. MZ showed a higher rank (22.50). However, BZ recorded the lowest mean rank at 12.50. The results also indicated a significant difference in chlorhexidine groups with an H value of 12.062, *p* = 0.002. The mean ranks of 23.30, 12.50 and 10.70 were recorded by LD, MZ and BZ, respectively. Table 3 shows the Mann-Whitney post hoc test between the groups. The results exhibited the statistically significant difference between all the restorative materials in coffee immersion, whereas in green tea immersion, the mean color change value difference between LD and BZ was statistically insignificant (*p* = 0.288). Additionally, chlorhexidine immersion also showed a similar trend with a statistically insignificant difference between glass ceramics and BZ with a *p* value of 0.579.

## 4. Discussion

The success of dental prostheses depends on their ability to restore function and aesthetics. Precisely matching the color to adjacent teeth along with other aesthetic objectives like position, texture and contour is vital for a good aesthetic outcome. Because of deeper translucency close to the natural tooth, all-ceramic restorations are preferred in the anterior region [22]. The permanency of the established color is decisive for the long-term success of prostheses. To interpret the effect of frequently consumed beverages like coffee, tea, and chlorhexidine mouth wash on the color stability of all-ceramic restoration, we tested these restorative material samples with a spectrophotometer. In the present study, the spectrophotometer was used for color measurement because they offer better accuracy, the numerical expression of color and are devoid of subjective bias [23,24]. Color in this study was expressed in CIELAB since it includes all perceivable colors and was developed to serve as a device-independent reference. The coordinate ‘L’ represents the lightness of color, while ‘a’ and ‘b’ denotes the chromatic range of green-magenta and blue-yellow, respectively. Because three coordinates are measured independently, it permits the measurement of infinitely many possible colors in three-dimensional real number space [25].

Based on the results of the present investigation, the null hypothesis of no discoloration of ceramic restoration with beverages and mouthwash was rejected. The surface disintegration of ceramics depends on material composition, fabrication methods, surface treatment and measurement methods [26,27] Coffee caused the least discoloration in pressed-glazed lithium disilicate e-max restorations. Palla et al. [28] reported that the rough surface of the unglazed pressed ceramic enables water infiltration and ensuant silica network disintegration. This leads to reduced crystallinity and enhanced absorption of coloring pigments. Whereas glazed-pressed ceramics, due to lack of surface irregularity and micro-cracks, prevent the water penetration and silica network dissolution. The conclusions from their study were in agreement with our study results. Gawriolek et al. [29] reported a similar mean color parameter of IPS e-max after soaking in the coffee for 72 hours at 1.71. Alencar-Silva et al. [30] described mean color change for both glazed and polished CAD-CAM lithium disilicate ceramic due to beverages is below the perceptibility threshold of 1.30.

The findings of our study indicated that monolithic zirconia was susceptible to higher discoloration from coffee, green tea and CXG. The corresponding ΔE values were 5.602, 5.192, and 4.866, respectively. These results are consistent with those of Kurt et al. [31], who showed that the color changes due to aging are higher in monolithic zirconia. They also found that the lithium disilicate ceramic was more aesthetic in terms of color stability and translucency. The monolithic zirconia, without an overlaying ceramic veneer, is directly exposed to the water and body fluids. The water exposure at 37 °C leads to low-temperature degradation (LTD) by phase transformation from a tetragonal to a monoclinic structure [32,33]. Phase transformation to monoclinic led to a 4% increase in volume; consequently results in structural disintegration, surface roughness and the development of micro-cracks [34]. Monolithic zirconia manufacturers reduce the alumina content to improve its translucency. Fathy et al. [35] propose that the alumina content is accountable for the resistance of low-temperature degradation. They reported that monolithic zirconia is more susceptible to LTD than core zirconia due to lower alumina content. Surface porosity because of monoclinic phase transformation also enhances the scattering of incident light and reduces the translucency [36]. 

The BZ with feldspar veneering ceramic showed the lesser discoloration across all the discoloring solution compared to monolithic zirconia. The corresponding ΔE values of coffee, green tea and chlorhexidine were 4.229, 2.11, and 1.438, respectively. The core Y-TZP was reported as being more resistant to LTD than MZ. Previous research has also shown that Core Y-TZP used in BZ prosthesis possesses higher crystal intensity counts compared to monolithic zirconia [37]. The superior resistance to LTD is also attributed to the smaller average crystal size within core Y-TZP. Keuper et al. [38] suggested that the larger grain sizes in monolithic zirconia are less resistant to the transformation, though they provide higher mechanical properties. The resultant micro-cracks from hydrothermal aging are not exposed to the discoloring solutions due to overlaying feldspar veneering ceramic. Enhanced refractive indices post hydrothermal aging is attributed to the increased monoclinic phase, crystallinity, grain size and porosity [39,40]. The reduced translucency of core Y-TZP could indirectly affect the resultant color of BZ. Suputtamongkol et al. [41] reported that the color of a background substructure affects the overall color of zirconia restorations. Camposilvan et al. [42] suggested the glaze as a barrier against hydrothermal aging for the underlying zirconia. Prior reports [43] have also shown that the surface texture of the finished restoration affects color stability, hence researchers advocate polishing or glazing to achieve a smoother surface and to improve the color stability. The mean color change observed with IPS e-max and BZ in the present study was less than 3.5. Previous research suggests that color changes (ΔE) less than 3.5 are indiscernible and clinically acceptable [44,45]. The monolithic zirconia showed mean color changes that were marginally higher than the clinically acceptable level.

The limitation of the present in vitro study includes the fact that the thermocycling was conducted in water, unlike saliva in the oral cavity. The staining solutions not being refreshed during the immersion period and the constant exposure of ceramics over 7 days of immersion could affect the discoloration of the tested ceramics. The complementary effects of brushing, micro-surface roughness and coloring agents in nutrition were not considered in the study. Additionally, the study did not include the effect of UV light on the discoloration process. Further studies are recommended to evaluate the effect of sunlight exposure, salivary proteins and nutritional coloring agent on the color stability of all-ceramic restorations.

## 5. Conclusions

Within the limitations of this in vitro study, the following conclusions were drawn:

(1) Discoloration from coffee was more significant in monolithic and bilayer zirconia compared to lithium disilicate ceramic. Green tea affected lithium disilicate more than coffee discoloration.
(2) Lithium disilicate ceramic was found to have better color stability compared to monolithic and bilayer zirconia.
(3) Monolithic zirconia displayed the least color stability among all the tested discoloring agents.

## Figures and Tables

**Table 1:** Mean ΔL, Δa, Δb and mean colour change (ΔE) values after immersion in staining solution for all ceramic restorations.

Restoration	Discoloring Solutions	ΔL	Δa	Δb	ΔE
Lithium disilicate ceramics	Coffee	−0.434	0.223	−1.00	1.788 (1.41)
	Green Tea	−1.583	−0.026	1.111	2.241 (1.35)
	Chlorhexidine	−0.155	0.179	0.135	1.588 (0.97)
Monolithic zirconia	Coffee	−3.560	0.877	3.493	5.602 (1.41)
	Green Tea	−0.918	0.837	2.525	5.192 (2.18)
	Chlorhexidine	−1.468	0.584	−1.475	4.866 (2.21)
Bilayer zirconia	Coffee	−2.690	1.546	2.165	4.229 (0.54)
	Green Tea	−0.259	0.187	−0.909	2.191 (2.18)
	Chlorhexidine	−1.228	0.302	−0.363	1.438 (0.89)

ΔL: Mean L (lightness); Δa: Mean a(green–red); Δb: Mean b (blue–yellow); ΔE: Mean Colour change.

**Table 2:** Kruskal-Wallis analysis of Mean color change between different all ceramic restorations.

Decoloring Solutions	Group	Mean Rank	ChiSquare	Df	*p* Value
Coffee	LD ceramics	6.10	19.886	2	0.000 *
	Monolithic zirconia	23.40			
	Bilayer Zirconia	17.00			
Green Tea	LD ceramics	13.40	10.025	2	0.007 *
	Monolithic zirconia	22.50			
	Bilayer Zirconia	10.60			
Chlorhexidine	LD ceramics	12.50	12.062	2	0.002 *
	Monolithic zirconia	23.30			
	Bilayer Zirconia	10.70			

Df: degrees of freedom, LD: Lithium disilicate; * The mean difference is significant at the 0.05 level.

**Table 3:** Mann-Whitney post hoc pairwise comparison between the mean color changes recorded in different groups.

Decoloring Solutions	Group	Glass Ceramic	Monolithic Zirconia	Bilayer Zirconia
Coffee	LD ceramics	-	0.000	0.000
	Monolithic zirconia	0.000	-	0.018
	Bilayer Zirconia	0.000	0.019	-
Green Tea	LD ceramics	-	0.008	0.288
	Monolithic zirconia	0.008	-	0.008
	Bilayer Zirconia	0.288	0.008	-
Chlorhexidine	LD ceramics	-	0.003	0.579
	Monolithic zirconia	0.003	-	0.002
	Bilayer Zirconia	0.579	0.002	-
